# Activities and participation of children and adolescents after mild traumatic brain injury and the effectiveness of an early intervention (Brains Ahead!): study protocol for a cohort study with a nested randomised controlled trial

**DOI:** 10.1186/s13063-016-1357-6

**Published:** 2016-05-06

**Authors:** M. Irene Renaud, Suzanne A. M. Lambregts, Arend J. de Kloet, Coriene E. Catsman-Berrevoets, Ingrid G. L. van de Port, Caroline M. van Heugten

**Affiliations:** Revant Rehabilitation Centre, Breda, The Netherlands; Department of Neuropsychology and Psychopharmacology, Maastricht University, Maastricht, The Netherlands; Department of Rehabilitation Medicine, Erasmus University Hospital/Sophia Children’s Hospital, Rotterdam, The Netherlands; The Hague University of Applied Sciences, Expertise Group Rehabilitation, The Hague, The Netherlands; Sophia Rehabilitation, The Hague, The Netherlands; Department of Paediatric Neurology, Erasmus University Hospital/Sophia Children’s Hospital, Rotterdam, The Netherlands; School for Mental Health and Neuroscience, Maastricht University, Maastricht, The Netherlands

**Keywords:** Activities, Participation, Mild traumatic brain injury, Children, Adolescents, Intervention, Study design, Randomised controlled trial

## Abstract

**Background:**

Approximately 20 % of children and adolescents who have sustained mild traumatic brain injuries may experience long-term consequences, including cognitive problems, post-traumatic stress symptoms and reduced load-bearing capacity. The underestimation and belated recognition of these long-term consequences may lead to chronic and disruptive problems, such as participation problems in school and in social relationships. The aim of this study is to examine the level of activities and participation of children and adolescents up to 6 months after a mild traumatic brain injury and to identify possible outcome predictors. Another aim is to investigate the effectiveness of an early psychoeducational intervention and compare the results with those obtained with usual care.

**Methods/design:**

This paper presents the Brains Ahead! study design, a randomised controlled trial nested within a multicentre, longitudinal, prospective cohort study. The eligible participants include children and adolescents between 6 and 18 years of age who have experienced a mild traumatic brain injury within the last 2 weeks. The cohort study will include 500 children and adolescents with a mild traumatic brain injury and their caregivers. A subset of 140 participants and their caregivers will be included in the randomised controlled trial. Participants in the randomised controlled trial will be randomly assigned to either the psychoeducational intervention group or the usual care control group. The psychoeducational intervention involves one face-to-face contact and one phone contact with the interventionist, during which the consequences of mild traumatic brain injury and advice for coping with these consequences to prevent long-term problems will be discussed. Information will be provided both verbally and in a booklet. The primary outcome domain is activities and participation, which will be evaluated using the Child and Adolescent Scale of Participation. Participants are evaluated 2 weeks, 3 months and 6 months after the mild traumatic brain injury.

**Discussion:**

The results of this study will provide insight into which children with mild traumatic brain injury are at risk for long-term participation problems and may benefit from a psychoeducational intervention.

**Trial registration:**

Netherlands Trial Register identifier NTR5153. Registered on 17 Apr 2015.

**Electronic supplementary material:**

The online version of this article (doi:10.1186/s13063-016-1357-6) contains supplementary material, which is available to authorized users.

## Background

The incidence of traumatic brain injury (TBI) in children between 0 and 18 years of age is 280–1373 per 100,000 but differs by country and region [1–8]. In the Netherlands, the annual estimated incidence of TBI among children and adolescents between ages 0 and 24 years is 5.86 per 1000 [9]. Therefore, approximately 12,000–14,000 cases of TBI occur among children and adolescents aged 0–24 years in the Netherlands each year, most (80 %) of which are mild traumatic brain injuries (MTBIs) [9, 10]. Children and adolescents with moderate and severe TBI generally receive follow-up care from a neurologist or rehabilitation physician, but those with MTBI typically do not [11, 12]. Notably, however, between 6 % and 43 % of children and adolescents with MTBI continue to experience symptoms 6 months after the injury and beyond [13–16]. MTBI in children and adolescents may lead to physical, cognitive, emotional and behaviour problems [17–19]. Several studies suggest that the post-concussive symptoms and cognitive deficits resulting from an MTBI resolve over time, but there is also evidence suggesting that these consequences persist in some children [20].

Previous studies of children who had experienced acquired brain injury (ABI) indicated that these children can also be at risk of participation limitations [21]. However, these studies often have included heterogeneous groups, making it difficult to identify the participation problems accompanying MTBI more specifically [21–24]. In addition to clarifying the long-term outcomes on the level of activities and participation, more research is needed on the predictors of outcome. The predictors of activity and participation outcomes following a childhood MTBI remain unclear [25–29]. Studies on overall outcome after a childhood MTBI suggest that both injury-related (e.g., Glasgow Coma Scale score, loss of consciousness, post-traumatic amnesia) and non-injury-related (e.g., age at injury, socioeconomic status, family functioning) factors affect outcome [30–34]. To determine which variables predict symptom resolution after an MTBI, well-designed, long-term studies are needed [20, 35].

Early recognition of symptoms and problems after an MTBI is crucial and enables the application of early and focused interventions [35, 36]. Long-term symptoms accompanying MTBIs, such as cognitive (e.g., attention) or behavioural symptoms, are often difficult to recognize or to associate with the MTBI [30]. Delayed recognition of these invisible symptoms, underestimation of these problems and delay of diagnosis frequently and unnecessarily lead to chronic and disruptive consequences, such as activity and participation limitations (e.g., in school and social relationships) [19, 37, 38]. Several studies indicate that early education, reassurance and even early cognitive behavioural approaches may be effective in preventing long-term problems after an ABI in both children and adults [39, 40], and, more specifically, after an MTBI [41–43].

Although the few available studies on interventions (e.g., psychoeducation) that prevent MTBI symptoms in children and adolescents have tended to report positive results, these studies have been retrospective or lacked a randomised controlled trial design [42–45]. The Brains Ahead! Study, using a randomised controlled trial and a large multicentre prospective cohort, is, to the authors’ knowledge, the first to examine the effect of a psychoeducational intervention on long-term activity and participation outcomes in children and adolescents who have experienced an MTBI.

The first aim of the Brains Ahead! study is to examine participation and activity levels in children and adolescents during the first 6 months after their MTBIs and to identify outcome predictors. We expect that 20 % of our study population will experience activity and participation problems during the first 6 months after their injuries [13–16, 20–24, 30, 36]. Furthermore, we hypothesize that injury-related and non-injury-related factors can predict outcomes [25–34].

The second aim is to investigate the effect of an early psychoeducational intervention on activities and participation. We hypothesize that, compared with usual care, our intervention will result in an increase in activities and participation during the first 6 months after an MTBI [39–45].

## Methods/design

### Study design

**Fig. 1:**
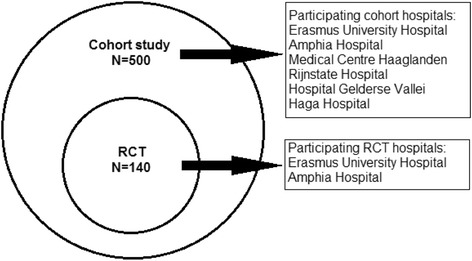
Study design. RCT, randomised controlled trial

**Fig. 2:**
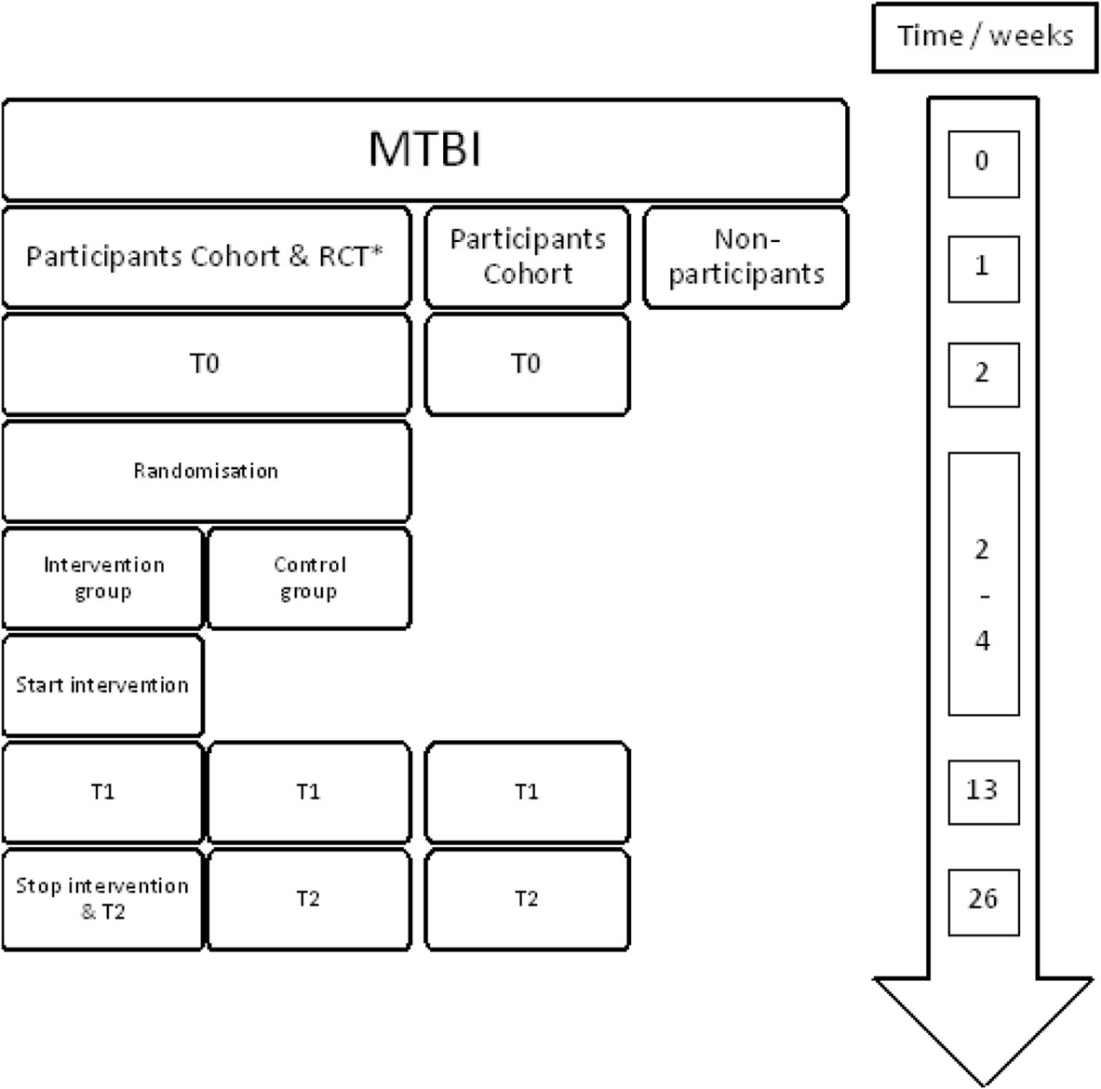
Flowchart of the study. *The randomised controlled trial (RCT) is performed in intervention hospitals only. MTBI, mild traumatic brain injury

**Fig. 3:**
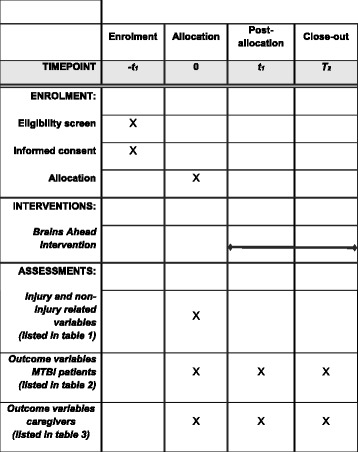
SPIRIT checklist. MTBI, mild traumatic brain injury

The study is a multicentre prospective longitudinal cohort study with a nested single-blind randomised controlled trial (RCT). The RCT is conducted using a subset of participants from the cohort study (Fig. 1) [46]. The protocol is described according to the Standard Protocol Items: Recommendations for Interventional Trials (SPIRIT) checklist for clinical trials (see Additional file 1 for the SPIRIT checklist). Participants are followed during the first 6 months post-injury. During this period, there are three measurement points: 2 weeks (T0), 3 months (T1) and 6 months (T2) post-MTBI (Figs. 2 and 3). The intervention begins 2–4 weeks post-injury and ends 6 months post-injury. The measurements and measurement times are the same for the cohort study and RCT participants. Measurements are performed by the researcher, who is blinded to the RCT group assignment.

### Study population

Participants are included at the emergency department (ED) of one of the six participating university and general hospitals in The Netherlands (Erasmus University Hospital, Rotterdam; Amphia Hospital, Breda; Medical Centre Haaglanden and Haga Hospital, The Hague; Rijnstate Hospital, Arnhem; and Hospital Gelderse Vallei, Ede). The subset of participants used for the RCT consists of participants from two of these six hospitals only (Fig. 1). To avoid selection bias, participants recruited from both a university hospital (Erasmus University Hospital, Rotterdam) and a large general city hospital (Amphia Hospital, Breda) will participate in the RCT. Participants will be recruited between April 2015 and December 2017. The medical ethics committee of Erasmus University Medical Centre, Rotterdam, and all of the local committees of the participating hospitals approved the study protocol (see the Additional file 2) (MEC-2015-047, NL51968.078.14, v03). The study is registered in the Netherlands Trial Register (NTR5153).

### Inclusion and exclusion criteria

The following inclusion criteria must be met to participate in the study: (1) children and adolescents aged 6–18 years and their caregivers (in this study, the caregiver is defined as a parent or guardian); (2) diagnosed with MTBI according to the criteria established by the American Congress of Rehabilitation Medicine and the World Health Organisation Collaborating Centre for Neurotrauma Task Force on Mild Traumatic Brain Injury [47] (p. 266); and (3) informed consent provided. All caregivers and all patients aged 12 years and older will provide written informed consent to participate in the cohort study, and caregivers and patients from the two RCT hospitals (Erasmus University Hospital and Amphia Hospital) will provide this for participation in the RCT as well. For children younger than 12 years of age, the caregiver will provide written consent. Exclusion criteria for children include having a previous objectified head trauma or having progressive neurological problems or diseases (based on patient history in the hospitals’ electronic patient files), attending a day-care or school for cognitively impaired children and youth, and having insufficient knowledge of Dutch (patient or caregiver).

### Patient selection and study procedures

In each of the six participating hospitals, all MTBIs are registered and communicated to the researcher. Within the first week after the MTBI, the researcher will contact the caregivers by phone to ask if they are willing to participate in the study. Subsequently, interested caregivers and patients receive written information about the study. There are two information sheets: one about the cohort study and one about the RCT. The last is only to be received by interested caregivers and patients from the two RCT hospitals. The baseline measurement (T0) is scheduled within 2 weeks post-injury and takes place at the participants’ home only after written informed consent is obtained by the researcher. Thereafter, the subset of participants from the two hospitals that participate in the RCT are randomised and the intervention group receives the intervention. Measurements take place at 3 months and 6 months post-injury and are equal for participants in the cohort study and in the RCT (see Figs. 2 and 3). The researcher is responsible for data management during the study. After the study is closed, data will be stored with the primary investigator.

### Randomisation procedures

Participants who agree to be included in the RCT are randomly assigned to either the intervention group or the control group. Randomisation is performed after the T0 measurement, which takes place within 2–4 weeks post-injury. It is performed by an independent person who is not involved in the recruitment, intervention or outcome measurements. The randomisation is performed using an online randomisation program that employs computerized block randomisation, and the randomisation scheme includes stratification based on three variables: age (6–12 years or 12–18 years), gender (male or female) and location (hospital). Caregivers are assigned to the same group as their child. After randomisation, the independent third person informs the interventionist (a professional experienced and educated in child rehabilitation after TBI) about the patients assigned to the intervention group, whereupon appointments for the intervention are scheduled.

### Intervention procedures

The intervention period begins 2–4 weeks post-injury and extends to 6 months post-injury. Optimally, the intervention is offered during the early phase of recovery to prevent long-term activity and participation problems. Two scheduled sessions occur during the intervention period. The first is a face-to-face session 2–4 weeks after the injury; the second is a telephone follow-up session 6–8 weeks after the injury. During the first 1-h face-to-face session, participants are screened for symptoms or trauma-related problems and receive individualized psychoeducation. The second session—the follow-up telephone call—will last approximately 30 minutes. Patients or their caregivers can also consult the interventionist when needed. After participants have received four or more optional follow-up sessions (or fewer, based on the clinical judgement of the interventionist), the patient and caregivers are advised to contact their general practitioner for evaluation or referral.

During the intervention period, there are no restrictions on obtaining care or treatment from other professionals. However, all participants are asked to complete a patient diary every month and record any care received. Information about the sessions (e.g., date, duration, content and whether more extensive information on certain topics is given) and the use of additional optional follow-up sessions (e.g., date, duration, content) are recorded during the intervention period by the interventionist. Furthermore, participants, caregivers and patients aged 12 years and older are individually asked to evaluate the intervention content and process at the end of the intervention.

### Content of the intervention

The intervention consists of the following content:

1. *Screening of symptoms and MTBI-related problems*: A list of the ten most frequently experienced post-injury symptoms and problems was developed by our research team.
2. *Psychoeducation*: The information provided during psychoeducation includes general information about symptom occurrence, possible symptoms and practical advice for managing symptoms and developing activities for children and adolescents with MTBI and their caregivers. It also includes more extensive individualized information about specific symptoms (e.g., headaches, dizziness and nausea, attention problems, memory problems). The general information about MTBI is provided verbally and in a written booklet. The booklet is available in three versions: a caregiver version, a version for patients aged 6–12 years and a version for patients aged 12–18 years. The individualized information is given only to participants who experience MTBI-related symptoms and is provided verbally and in writing.
3. *Follow-up*: A single follow-up is held via telephone. Depending on the needs of the patient or caregiver, optional additional follow-up telephone sessions may be scheduled.

The control group receives usual care. Each hospital has a concise standard information brochure that briefly describes the possible consequences of MTBI and what to do if MTBI symptoms persist and increase. This brochure is usually given to patients in the ED.

### Outcome measurements

Several injury-related and non-injury-related variables are identified. These variables are presented in Table 1 and Fig. 3.

**Table 1:** Injury/non-injury-related variables

Injury-related variables
Glasgow Coma Scale score (first recorded in the ambulance or ED)
Post-traumatic amnesia duration in minutes
Loss of consciousness reported in ED
Change in mental functioning: post-acute confusion or disorientation
Other transient neurological abnormalities
CT/MRI/EEG abnormalities
Cause of MTBI
Non-injury-related variables
Location (hospital where MTBI was diagnosed)
Admission to hospital
Age of patient at injury
Gender
Education level of patient
Pre-injury behavioural and emotional problems of the patient (measured using the CBCL)
Parental socioeconomic status
Pre-injury family function (measured using the FAD-GF)
Family situation (number of family members residing with the patient)

*ED* emergency department, *CBCL* Child Behaviour Checklist, *FAD-GF* Family Assessment Device–General Functioning Scale

The instruments used to measure activity and participation after an MTBI and possible outcome predictors are presented in Fig. 3, as well as in Table 2 for patients and in Table 3 for caregivers, and are described in more detail hereafter. All instruments described below are completed based on post-injury functioning, unless stated otherwise. Given the fact that a subset of the cohort sample will receive the intervention, this might influence the outcome data in the cohort study. Therefore, if the intervention is found to be effective, the outcome data of the intervention group will be excluded from all cohort analyses (see Statistical analyses subsection below).

**Table 2:** Outcome domains, measurement instruments and measurement moments for the patients with mild traumatic brain injury

Domain	Measurement instrument	Abbreviation	Age (years)	T0	T1	T2
Activities and participation	Children’s Assessment of Participation and Enjoyment	CAPE	6–18	X	X	X
	Child and Adolescent Scale of Participation–Dutch language version	CASP-DLV	10–18	X	X	X
Quality of life	PedsQL–Quality of Life Scale	PedsQL-QoL	6–18	X		X
Fatigue	PedsQL–Multidimensional Fatigue Scale	PedsQL-Fatigue	6–18	X		X
Health and behaviour	Health Behaviour Inventory	HBI	8–18	X		X
Post-traumatic stress	Schokverwerkingslijst (Impact of Event Scale–Dutch language version)	SVL (IES)	8–18	X		X
Sensory processing	Adolescent Adult Sensory Profile–Dutch version	AASP-NL	12–18	X		X

*PedsQL* Paediatric Quality of Life Inventory, *T0* 2 weeks after mild traumatic brain injury (MTBI), *T1* 3 months after MTBI, *T2* 6 months after MTBI

**Table 3:** Outcome domains, measurement instruments and measurement moments for the caregivers

Domain	Measurement instrument	Abbreviation	Age (years)	T0	T1	T2
Activities and participation^a^	Child and Adolescent Scale of Participation–Dutch language version	CASP-DLV	All	X	X	X
Quality of Life	PedsQL–Quality of Life Scale	PedsQL-QoL	All	X		X
Fatigue	PedsQL–Multidimensional Fatigue Scale	PedsQL-Fatigue	All	X		X
Health and behaviour	Health Behaviour Inventory	HBI	All	X		X
Post-traumatic stress	Schokverwerkingslijst (Impact of Event Scale–Dutch language version)	SVL (IES)	All	X		X
Family functioning	Family Assessment Device–General Functioning Scale	FAD-GF	All	X		X
Behaviour and emotion	Child Behaviour Checklist	CBCL	All	X		X
Sensory processing	Sensory Profile–Dutch short version	SP-NL	6–11	X		X

*PedsQL* Paediatric Quality of Life Inventory, *T0* 2 weeks after mild traumatic brain injury (MTBI), *T1* 3 months after MTBI, *T2* 6 months after MTBI

^a^Primary outcome measure

#### Primary outcome measure

The primary outcome measure, the Child and Adolescent Scale of Participation (CASP), is based on the activity and participation components of the International Classification of Functioning, Disability and Health for Children and Youth (ICF-CY). The CASP–Dutch language version (CASP-DLV) is a 20-item questionnaire designed specifically to measure activities and participation in children who have experienced an ABI [9]. It includes a parent-report and a self-report version for children aged 10 years and older. Our primary outcome will be limited to the results of the parent-reports. The CASP-DLV items are categorized into four domains: (1) participation at home, (2) participation in the district and residence, (3) participation at school, and (4) participation at home and in the environment. The questionnaire has been used in several international studies and has been recommended as an instrument for evaluating participation in children and adolescents after brain injury [34]. The internal consistency (Cronbach’s α 0.95) and test-retest reliability (intra-class correlation coefficient 0.90) of the CASP-DLV were found to be good and to have a significant correlation with the Paediatric Quality of Life Inventory (PedsQL) (concurrent validity 0.45) [48].

#### Secondary outcome measures

##### Child and Adolescent Scale of Participation–Dutch language version self-report

The CASP-DLV self-report questionnaire for children aged 10–18 years is used as a secondary outcome measure. It evaluates participation after an MTBI from the child’s perspective. The self-report version includes the same items and domains as the CASP-DLV parent-report. The self-report (or youth-report) of the original CASP is a psychometrically adequate self-report instrument for measuring activity and participation (internal consistency Cronbach’s α 0.87 and strong internal structure validity). It is used in conjunction with the CASP-DLV parent version because children and adolescents may have different perceptions than their parents about their activity and participation levels [49]. For children between the ages of 6 and 9 years, however, only the parent version is used. Information about participation from the child’s perspective is obtained using the Children’s Assessment of Participation and Enjoyment (CAPE).

##### Children’s Assessment of Participation and Enjoyment

The CAPE is a 55-item questionnaire whose items correspond to 55 different activities. It measures children’s participation in after-school activities [50, 51]. Five domains of participation are included: (1) diversity, (2) intensity, (3) setting and/or with whom the activity is typically performed, (4) usual location of the activity and (5) the amount of pleasure the child experiences during the activity. A comparison between the CAPE and the CASP-DLV parent version showed no significant correlation, which may be due to the difference in focus of the two questionnaires: one is focused on activity restriction and the other on diversity and intensity of participation [48]. The CAPE is found to be sensitive over time when measuring functional change in children after an MTBI [27]. Furthermore, the CAPE is also a reliable and valid tool for measuring participation in recreation and leisure activities in Dutch children aged 6–18 years with and without physical disabilities [51].

##### Paediatric Quality of Life Inventory–Quality of Life Scale

The Paediatric Quality of Life inventory–Quality of Life Scale (PedsQL-QoL) is a 23-item questionnaire that measures health and activities, emotions, peer relationships and school-related activities [52]. The questionnaire is internationally recommended for studies of children and adolescents who have experienced an ABI [25]. The psychometric properties of the Dutch PedsQL are found to be adequate, and the questionnaire is appropriate for paediatric research on health-related quality of life in the Netherlands [52].

##### Paediatric Quality of Life Inventory–Multidimensional Fatigue Scale

The Paediatric Quality of Life Inventory–Multidimensional Fatigue Scale (PedsQL-Fatigue) is an 18-item questionnaire that measures overall fatigue, problems regarding sleep and/or rest, and cognitive fatigue [53]. This questionnaire is recommended for studies of children and adolescents after an ABI [25]. The feasibility, reliability and validity of the Dutch version of the PedsQL–Multidimensional Fatigue Scale are adequate, and the scale distinguishes healthy children from children with an impaired health condition [53].

##### Health Behaviour Inventory

The Health Behaviour Inventory (HBI) is a 50-item questionnaire. It measures (1) physical, (2) emotional, (3) cognitive and (4) behavioural symptoms. The HBI has sound psychometric properties and is able to distinguish MTBI from other injuries [25, 54]. Because a Dutch version of this inventory did not yet exist, we translated the original HBI into Dutch according to the translation guidelines [55].

##### Impact of Event Scale

The Dutch version of the Impact of Event Scale (IES-NL) is a 34-item questionnaire that measures possible post-traumatic stress responses [56]. The items are divided into four dimensions: (1) re-experiencing the stressor, (2) avoidance, (3) increased irritability and (4) child-specific responses. The IES-NL has adequate reliability across various traumatic stressors and reveals a robust structure over various samples [56]. Furthermore, the questionnaire is internationally recommended for studies of children and adolescents who have experienced an ABI [25].

##### Family Assessment Device–General Functioning Scale

The Family Assessment Device–General Functioning Scale (FAD-GF) is a 12-item questionnaire used to measure family functioning. It has been used in previous studies on brain injuries in children [31] and is recommended for studies of pre-injury family problems and changes in family functioning associated with the traumatic brain injury [25, 57, 58]. The psychometric properties of the FAD-GF are sufficient for assessing family functioning [59]. This questionnaire is used to evaluate pre-injury family functioning at T0 and post-injury family functioning at T2.

##### Child Behaviour Checklist

The Child Behaviour Checklist (CBCL) is a 113-item questionnaire widely used to measure behavioural and emotional problems and skills in children [60]. This questionnaire is recommended for examining these problems in children and adolescents who have experienced an ABI and has sound psychometric properties [25, 60]. It is used to assess pre-injury behaviour and emotional problems and skills at T0 and post-injury behavioural and emotional problems and skills at T2.

##### Sensory Profile–Dutch short version and Adolescent/Adult Sensory Profile–Dutch version

The Sensory Profile–Dutch short version (SP-NL) is a 38-item questionnaire. In this study, it is completed by the parents of patients between 6 and 11 years old. Patients aged 12 years and older complete the Adolescent/Adult Sensory Profile–Dutch version (AASP-NL). The questionnaires measure sensory information processing—including several sensory functions, movement abilities and social-emotional aspects—and assess the child’s activity and participation levels [61, 62]. The questionnaire adequately measures sensory information processing in children after a traumatic brain injury [63].

### Sample size

Sample size calculations for the cohort study are based on the available literature about MTBI prevalence and the expected number of participants who may visit the participating hospitals. Based on an inclusion period of 2 years, the aim is to recruit a sample of 500 children and adolescents who have experienced an MTBI. Assuming a 10 % dropout rate [64], our final sample should include 450 participants. Previous research shows that approximately 20 % of the population will experience long-term problems [13–16, 20–24, 30, 36] after an MTBI. Therefore, approximately 90 of our participants will experience long-term problems. When conducting the regression analysis to identify the predictors of the presence of long-term problems, we should include nine determinants, based on the assumption that approximately ten participants per determinant are needed for a reliable analysis [65].

Sample size calculations for the RCT are based on the results of studies on paediatric traumatic brain injury patients’ participation that relied on the parent-reports of the CASP-DLV. For the CASP-DLV, a standardized difference of 0.5 was expected [48]. With an α of 0.05 and a power of 0.8, a minimum of 63 children per group (control group and intervention group) is required for sufficient statistical power. Assuming a dropout rate of 10 %, the aim is to recruit at least 140 children and adolescents for the RCT.

### Statistical analyses

Descriptive statistics will be used to present the data on the participants, number of dropouts, losses during follow-up and the outcome measure scores. To determine the sample’s representativeness and the generalizability of the results, participants will be compared with non-participants based on the inclusion and exclusion criteria. Furthermore, the baseline characteristics of participants and dropouts, as well as patients lost during follow-up, will be compared. Comparisons will be performed using independent samples *t* tests or the non-parametric equivalent.

#### Cohort study

To determine the results of the primary outcome measure (CASP-DLV parent-reports), descriptive statistics will be used. Continuous variables will be expressed as the means and standard deviations or as medians with interquartile ranges, depending on the distribution values. Repeated-measures analysis of variance will be used to determine the difference in activities and participation over time. If a significant difference between the measurement points (*p* < 0.05) is found, a post hoc analysis based on Levene’s test will be performed.

Linear regression analysis will be used to identify the outcome predictors of activities and participation at 6 months post-injury, as measured by the CASP-DLV parent reports. Within 2 weeks after the injury, both continuous and categorical variables (i.e., injury and non-injury-related factors) are measured, as well as pre-injury family functioning (FAD-GF) and behaviour (CBCL); degree of fatigue (PedsQL-Fatigue); quality of life (PedsQL-QoL); sensory processing (SP/AASP-NL); physical, cognitive, emotional and behavioural post-concussive symptoms (HBI); post-traumatic stress (Schokverwerkingslijst [Impact of Event Scale–Dutch language version]); and participation in after-school activities (CAPE). Each variable will first be examined using univariate linear regression analysis to predict activities and participation. Next, variables with values of *p* < 0.2 in the univariate linear regression analysis will be included in the multivariate linear regression analysis. In the multivariate linear analysis, the significance level will be set at *p* < 0.05. For more clinically relevant purposes, outcome predictors will also be determined using logistic regression analyses. If the intervention is found to be effective (see statistical analyses of RCT study below), the data of the intervention group will be excluded from all of the cohort study analyses.

#### RCT

First, the baseline characteristics of the two groups will be examined using independent samples *t* tests or Mann-Whitney *U* tests (depending on the distribution values). A χ^2^ test will be used to examine dichotomous variables. Next, the effectiveness of the intervention on the primary outcome measure (CASP-DLV parent-reports) will be assessed using multilevel analysis (i.e., random coefficient analysis) for both short-term (3 months after injury) and long-term (6 months after injury) outcomes. Time of measurement, group assignment (control or intervention group) and the interaction between time of measurement and group will be included in the multi-level regression model. The level of significance will be *p* < 0.05. The random coefficient analysis will be performed with all of the participants using intention-to-treat analyses. For those with incomplete datasets, longitudinal imputation techniques will be used [66].

## Discussion

This paper describes the research protocol of the Brains Ahead! study. The study examines the activities and participation outcomes of children and adolescents during the first 6 months after experiencing an MTBI and identifies possible outcome predictors. Furthermore, this study investigates the effectiveness of an early psychoeducational intervention on activities and participation compared with the usual MTBI care received by this population. We chose a nested design because it is preferred to gain insight into the effect of the intervention on a short-term basis, since it might help to prevent long-term problems after MTBI in children and adolescents. In this study, a large sample is recruited for the cohort part. Taking a subset of these participants for the RCT along at the same time enables us to investigate the effectiveness of the intervention faster than waiting on the results of the cohort study first and setting up a new intervention study afterwards. We believe this is an efficient way of investigating this group of participants from an ethical perspective as well. In many studies, various types of TBI (mild, moderate, severe) are included. However, this study investigates activities and participation in children and adolescents with MTBI only. In a study by Ponsford et al. [42], the effectiveness of an early intervention in the form of a general information booklet was evaluated in a mild paediatric population only. However, their study measured the impact of the intervention on reported symptoms, cognitive performance and psychological adjustment and not on preventing activity and participation problems. Furthermore, the sample size of their study was small (*N* = 61) compared with the expected sample size of the present study, and the outcome was measured at 3 months post-injury, while this study measures the outcome at 3 months and 6 months post-injury. The strength of this study is the substantial RCT sample size extracted from a large cohort. Furthermore, the outcome instruments used in this study are based largely on the ICF-CY.

To the authors’ knowledge, this is the first study to examine the effect of an early individualized psychoeducational intervention designed to prevent activity and participation problems in a relatively large group of children and adolescents following an MTBI. All of the participants in the nested RCT design receive usual care, and the intervention group receives an additional intervention. The intervention has a specific theoretical basis, and its design is based on evidence from the literature. Finally, and perhaps most importantly, the intervention is created to suit clinical practice and can be applied easily and directly in daily practice after its effectiveness has been proven. The results of this study will provide insight into which children with MTBI are at risk for long-term participation problems and may benefit from a psychoeducational intervention.

### Trial status

The trial is still ongoing. The first participant was included in May 2015. The planned closing date is December 2017.

## Additional files

Additional file 1: SPIRIT 2013 Checklist: recommended items to address in a clinical trial protocol and related documents. (DOC 124 kb) Additional file 2: Local committees that approved the study. (DOCX 13 kb)

## Abbreviations

AASP-NL: Adolescent/Adult Sensory Profile–Dutch version
ABI: acquired brain injury
CAPE: Children’s Assessment of Participation and Enjoyment
CASP: Child and Adolescent Scale of Participation
CASP-DLV: Child and Adolescent Scale of Participation–Dutch language version
CBCL: Child Behaviour Checklist
CT: computed tomography
ED: emergency department
EEG: electroencephalography
FAD-GF: Family Assessment Device–General Functioning Scale
HBI: Health Behaviour Inventory
ICF-CY: International Classification of Functioning, Disability and Health for Children and Youth
IES: Impact of Event Scale
MRI: magnetic resonance imaging
MTBI: mild traumatic brain injury
PedsQL: Paediatric Quality of Life Inventory
PedsQL-QoL: Paediatric Quality of Life Inventory–Quality of Life Scale
RCT: randomised controlled trial
SP-NL: Sensory Profile–Dutch version
SPIRIT: Standard Protocol Items: Recommendations for Interventional Trials
TBI: traumatic brain injury
