# Supplementary material for: Activities and participation of children and adolescents after mild traumatic brain injury and the effectiveness of an early intervention (Brains Ahead!): study protocol for a cohort study with a nested randomised controlled trial
Source: Trials. 2016 May 6;17:236. doi: 10.1186/s13063-016-1357-6 (PMC4858836; doi:10.1186/s13063-016-1357-6)
Supplement: Additional file 2: — Local committees that approved the study. (DOCX 13 kb) [file 13063_2016_1357_MOESM2_ESM.docx]

Additional file 2

The additional file of local committees that approved the study

The Medical Ethics Committee of Erasmus University Medical Centre, Rotterdam approved the study protocol (MEC-2015-047, NL51968.078.14, v03).

Erasmus University Hospital, Rotterdam:
Head of the Department of Neurology, prof. dr. P.A.E. Sillevis Smitt

Hospital Gelderse Vallei, Ede:
Executive Board (in Dutch: Raad van Bestuur) after positive advice of the locoal Beoordelings-Commissie Wetenschappelijk Onderzoek (BCWO)

Amphia Hospital, Breda:
Executive Committee (in Dutch: Directiecomité) after positive advice of the local Adviescommissie Mensgebonden Onderzoek Amphia (AMOA)

Medical Centre Haaglanden:
Executive Board (in Dutch: Raad van Bestuur)

Rijnstate Hospital:
Executive Board (in Dutch: Raad van Bestuur) after positive advice of the Lokale Haalbaarheidscommissie (LHC)

Haga Hospital:
Executive Board (in Dutch: Raad van Bestuur)
